# Cell-free enzymatic synthesis of GDP-l-fucose from mannose

**DOI:** 10.1186/s13568-019-0798-1

**Published:** 2019-05-27

**Authors:** Weiyang Wang, Fan Zhang, Yanyun Wen, Yanbo Hu, Ye Yuan, Min Wei, Yifa Zhou

**Affiliations:** Jilin Province Key Laboratory on Chemistry and Biology of Changbai Mountain Natural Drugs, School of Life Sciences, Northeast Normal University, Changchun, 130024 People’s Republic of China

**Keywords:** GDP-l-fucose, Enzymatic synthesis, Mannose-6-phosphate, GDP-d-mannose

## Abstract

GDP-l-fucose, the key substrate for fucosyloligosaccharide biosynthesis, has been synthesized via a de novo pathway in bacteria. In the present study, genes for GDP-l-fucose biosynthesis were cloned into the expression vector pET-28a (+) to construct five *E. coli* strains, with recombinant enzymes being purified by using Ni–NTA chromatography. Following optimization of the 3-step reaction, Glk, ManB and ManC were added to the reaction mixture, after which Gmd and WcaG were added to overcome feedback inhibition from the end-product to produce GDP-l-fucose at 178.6 mg/l, with a yield of 14.1%. Our studies provide the basis for using cell-free enzyme production of GDP-l-fucose.

## Introduction

In human milk, fucosyloligosaccharide activates numerous biological processes, like prevention of infections, probiotic growth, and an improved immune response, in particular for infantile enteric and other pathogenic effects (Kunz and Rudloff 2006; Boehm and Stahl 2007; Chaturvedi et al. 2001). GDP-l-fucose is an essential precursor for the biosynthesis of fucosyloligosaccharide, providing fucosyl groups to oligosaccharides (Sun et al. 1995; Satoshi 2003). GDP-l-fucose is one of the most expensive and rare nucleotide sugars. Currently it is made by using a relatively expensive chemical synthetic protocol, which is insufficient to meet demand for large scale industrial production (Khaled et al. 2004).

**Fig. 1:**
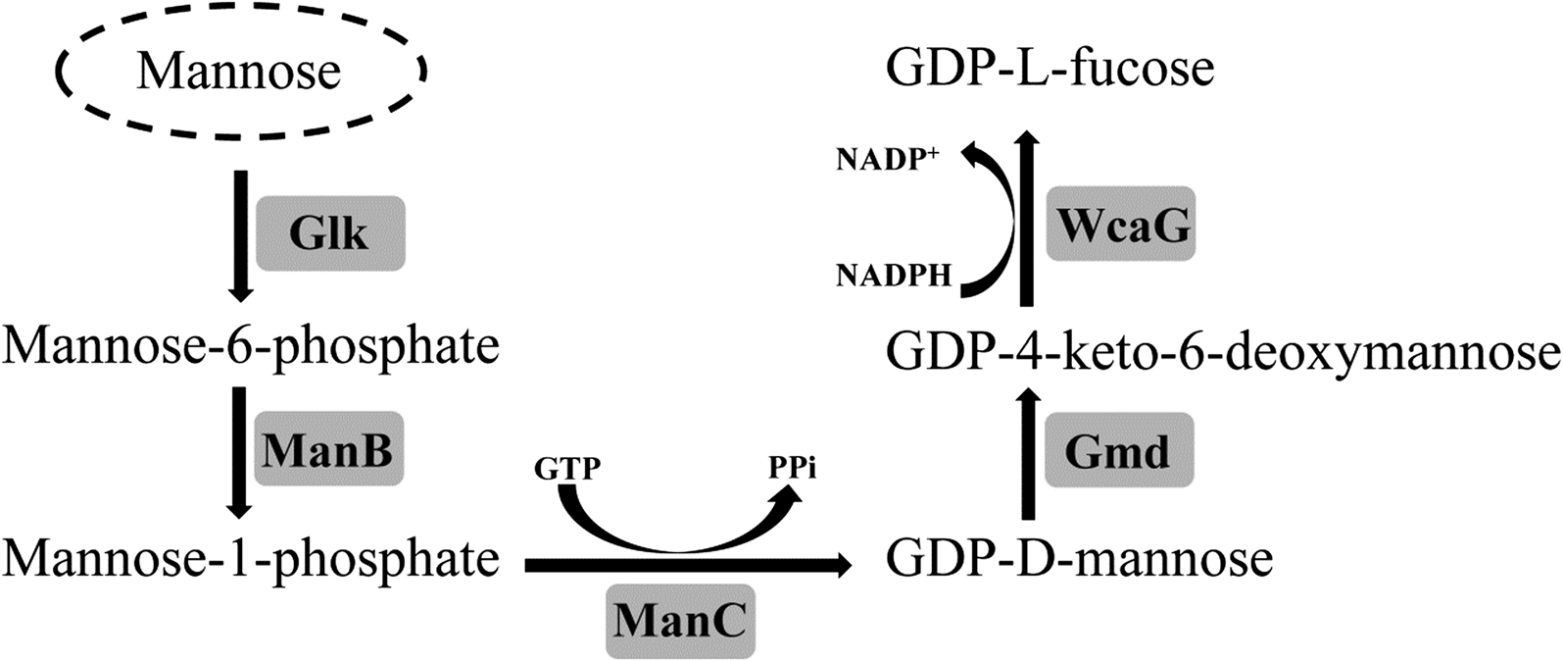
De novo biosynthetic pathway of GDP-l-fucose. *Glk* glucokinase, *ManB* phosphomannomutase, *ManC* mannose-1-phosphate guanyltransferase, *Gmd* GDP-d-mannose-4,6-dehydratase, *WcaG* GDP-4-keto-6-deoxymannose-3,5-epimerase-4-reductase

In vivo, GDP-l-fucose is generated by two pathways: the major de novo metabolic pathway and the minor salvage metabolic pathway (Becker and Lowe 2003; Niittymäki et al. 2004). The de novo pathway in bacteria, mammals and plants, starts from d-mannose conversion into GDP-l-fucose via a 5-step reaction (Fig. 1) (Chin et al. 2013). The initial step uses glucokinase (Glk, EC 2.7.1.63) to catalyze conversion of d-mannose to mannose-6-phosphate, that is then transformed to mannose-1-phosphate by phosphomannomutase (ManB, EC 5.4.2.8). Mannose-1-phosphate is then transformed to GDP-d-mannose by mannose-1-phosphate guanyltransferase (ManC, EC 2.7.7.13). GDP-d-mannose is processed to GDP-l-fucose in two additional steps: GDP-d-mannose-4,6-dehydratase (Gmd, EC 4.2.1.47) converts GDP-d-mannose to GDP-4-keto-6-deoxymannose and GDP-4-keto-6-deoxymannose-3, 5-epimerase-4-reductase (WcaG, EC 1.1.1.271) produces the end product, GDP-l-fucose.

Based on this well-studied pathway, many fermentation processes produce GDP-l-fucose using inexpensive and readily available mannose, have been developed. *Corynebacterium* sp. and *E. coli* are the primary producers of GDP-l-fucose. One approach to increase GDP-l-fucose production is to overexpress GDP-l-fucose genes and optimize fermentation (Koizumi et al. 2000; Byun et al. 2007; Chin et al. 2016; Albermann et al. 2000). Alternatively, one can combine engineered *E. coli* and *Corynebacterium* sp., with *E. coli* being used to overexpress GDP-l-fucose biosynthetic genes and *Corynebacterium* sp. to generate GTP and NADPH (Lee et al. 2011, 2012). However, there are drawbacks to microbial fermentation, such as bacterial growth status, complicated separations, and the presence of by-products (Qin et al. 2016). As an alternative, enzymatic synthesis provides greater efficiency by specifying GDP-l-fucose enzymes that can be immobilized and reused, thus lowering production costs and limiting the amounts of by-products (Liu et al. 2015). As far as we know, there is no report on cell-free enzymatic synthesis of GDP-l-fucose from mannose.

In the present study, *E. coli* strain carrying individual expression cassettes of the de novo pathway biosynthesis genes was constructed. After improving protein yield and purifying enzymes, we optimized enzymatic synthesis of GDP-l-fucose. Our studies provide a basis for the immobilization of multi-enzyme complexes and pave the way for industrial GDP-l-fucose production.

## Materials and methods

### Cloning and expression of the targeted enzymes

The nucleotide sequence of the 807 base pair fragment encoding the Glk gene (CP000518) from *Mycobacterium sp*. (strain MCS) was synthesized by Synbio Technologies Inc. (Jiangsu, China). The genes that encode ManC, Gmd and WcaG were PCR amplified from the DNA of *E. coli* BL21 (DE3). The manB gene was amplified by using PCR and the *E. coli* K12 strain ER3413 (*E. coli* Genetic Stock Center; CGSC#14167) DNA as the template. Cloning primer sequences were designed according to GenBank (manB: CP032667, manC: CP020368, gmd: CP010816, wcaG: CP001665) as follows:

ManB: 5′-CGGGATCCATGAAAAAATTAACCTGCTTTAAA-3′ and 5′-CCCTCGAGTTACTCGTTCAGCAACGTCAGCAG-3′; ManC: 5′-CGGGATCCATGGCGCAGTCGAAACTCTATCCA-3′ and 5′-CGGAATTCTTACACCCGTCCGTAGCGATCCGC-3′; Gmd: 5′-CGGAATTCATGTCAAAAGTCGCTCTCATCACC-3′ and 5′-GCGTCGACTTATGACTCCAGCGCGATCGCCAC-3′; WcaG: 5′-CGGGATCCATGAGTAAACAACGAGTTTTTATT-3′ and 5′-CCCTCGAGTTACCCCCGAAAGCGGTCTTGATT-3′.

These five genes were cloned into the pET-28a (+) vector with the T7 promoter, and the recombinant plasmid was transfected into *E. coli* BL21 (DE3). A single colony of *E. coli* BL21 (DE3) carrying the recombinant plasmid was inoculated in 10 ml Luria–Bertani (LB) culture medium with 0.1 mg/ml kanamycin and grown at 37 °C. The overnight-grown culture was diluted using 50 ml terrific broth (TB) at 37 °C, followed by IPTG addition of to induce protein expression. This was done when the cell density was OD_600_ 0.5–0.6, and the culture was incubated at 25 °C with agitation.

### Purification of recombinant enzymes

The intracellular soluble fraction was centrifuged at 12,000×*g* for 15 min to remove insoluble cell debris after cells were harvested and disrupted by sonication. Ammonium sulfate (20% w/v) was added to the cell-free sample extract, and the precipitate was collected and dissolved in phosphate buffer and dialyzed against phosphate buffer (2 l) at 4 °C overnight. The sample was filtered with 0.22 μm filters (Jiangsu Green Union Science Instrument Co., Ltd.), and loaded on a Ni–NTA column. Fractions containing the target enzyme were combined and dialyzed against phosphate buffer (2 l) at 4 °C. Proteins were monitored by using sodium dodecyl sulfate–polyacrylamide gel electrophoresis (SDS-PAGE), and concentrations were determined by using the method of Bradford (Bradford 1976).

### Production and determination of mannose-6-phosphate

The reaction mixture (5 mM mannose, 10 mM GTP and 0.75 mg/ml Glk in PBS buffer) was incubated at 37 °C for 10 h. Reaction mixtures were incubated at 100 °C for 5 min to inactivate enzyme, and samples were centrifuged at 12,000×*g* for 10 min and analyzed by using HPAEC. HPAEC-PAD analysis was performed using a Dionex ICS 5000 system with a CarboPac PA-200 column and pulsed electrochemical detector. Samples were eluted at 0.5 ml/min with 2 mM NaAc for 5 min, and then by using a linear gradient from 2 mM to 300 mM NaAc in 200 mM NaOH for 20 min.

### Production and Determination of GDP-d-mannose

Reactions were run at 37 °C using a mixture of 2.5 mM mannose, 5 mM GTP, 2.5 mM MgCl_2_, 1 mg/ml ManB, 0.38 mg/ml Glk, 10 μM glucose-1, 6-bisphosphate and 1 mg/ml ManC in PBS buffer. Following incubation for 12 h, mixtures were incubated at 100 °C for 5 min, and centrifuged at 12,000×*g* for 15 min. GDP-d-mannose was detected by using a Q Exactive quadrupole Orbitrap mass spectrometer (Thermo Scientific) in the negative ion mode. Operating parameters were as follows: sample was injected into the peristaltic pump with a flow rate of 20 μl/min; maximum inject time of 50 ms; spray voltage, 3.0 kV; sheath gas pressure, 15 psi; auxiliary gas pressure, 2 arb; capillary temperature, 320 °C; scan type: full mass; resolution, 70,000; scan range, m/z 150–1000; scan range, 100–1000. GDP-d-mannose content was analysed by HPLC (Shimadzu LC-10 AT VP) equipped with strong anion exchange column (4.6 × 250 mm). The mobile phase consisted of 90% potassium dihydrogen phosphate (0.1 M, pH 3.5) and 10% acetonitrile (v/v) used at a flow rate of 0.7 ml/min. The reaction products were detected by UV absorbance at 260 nm.

### Production and determination of GDP-l-fucose

Briefly, 3-step reaction was performed as follows: (1) The reaction mixture (2.5 mM mannose, 10 μM glucose-1,6-bisphosphate, 2.5 mM MgCl_2_, 0.38 mg/ml Glk, 5 mM GTP, 1 mg/ml ManB and 1 mg/ml ManC in PBS buffer) was incubated at 37 °C for 12 h. Samples were incubated at 100 °C for 5 min and centrifuged at 12,000×*g* for 10 min. 2) NADP^+^ (0.1 mM), MgCl_2_ (0.1 mM) and Gmd (1 mg/ml) were added to the sample, and the sample was incubated at 37 °C for 2 h. Heat-inactivated enzymes were removed by centrifugation for 10 min at 12,000×*g*. (3) NADPH (1 mM) and WcaG (1 mg/ml) were added to samples that were then incubated at 37 °C for 2 h. Analysis of GDP-l-fucose was carried out using HPLC (Shimadzu LC-10 AT VP) equipped with strong anion exchange column (4.6 × 250 mm). The mobile phase consisted of 90% potassium dihydrogen phosphate (0.1 M, pH 3.5) and 10% acetonitrile (v/v) used at a flow rate of 0.7 ml/min. The reaction products were detected by UV absorbance at 260 nm.

### Amino acid sequences

GenBank accession number: Glk: ABL91430; ManB: AYG18923; ManC: ARH97725; GMD: AJH10789; WcaG: ACT28661.

## Results

### Overexpression and purification of recombinant enzymes

**Fig. 2:**
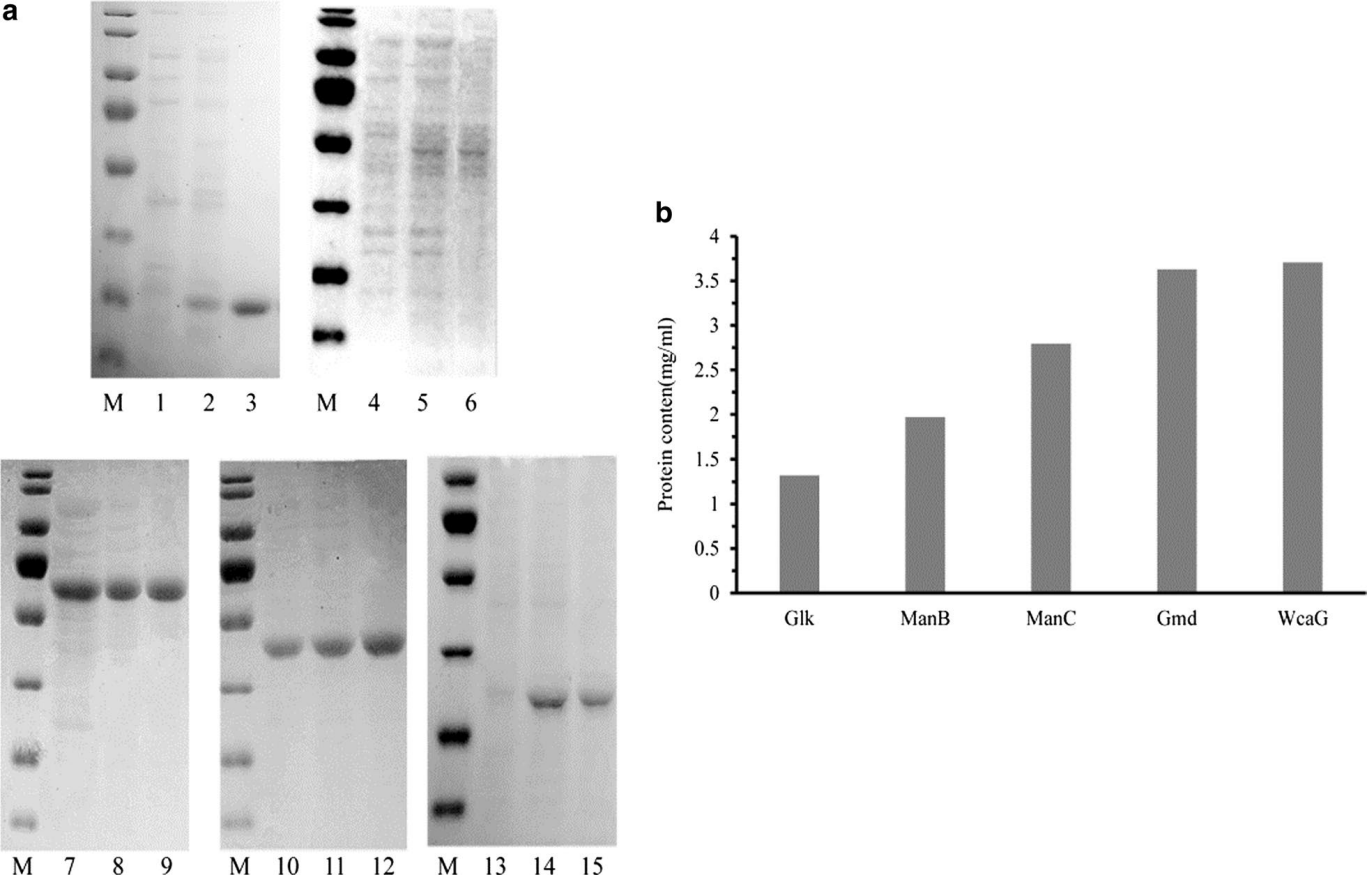
**a** SDS-PAGE analysis of the recombinant GDP-l-fucose biosynthetic enzymes. Lane M: molecular weight marker; Lane 1–3: Glk (−IPTG), Glk (+IPTG), purified Glk; Lane 4–6: ManB (−IPTG), ManB (+IPTG), purified ManB; Lane 7–9: ManC (−IPTG), ManC (+IPTG), purified ManC; Lane 10–12: Gmd (−IPTG), Gmd (+IPTG), purified Gmd; Lane 13–15: WcaG (−IPTG), WcaG (+IPTG), purified WcaG. **b** Protein concentrations of recombinant enzymes

Genes encoding ManC, Gmd and WcaG were PCR-amplified from *E. coli* BL21 DNA. The manB gene was amplified by using PCR with *E. coli* K12 strain ER3413 DNA as template. PCR products, as well as the synthesized gene product (Glk) were sub-cloned into T7-driven expression vector pET-28a (+), and recombinant plasmids were transfected into *E. coli* BL21 (DE3). The engineered *E. coli* strain was grown in Terrific Broth (TB) medium, and IPTG was added to induce protein expression. His-tagged fusion proteins were purified by using the ammonium sulfate precipitated fraction, followed by Ni–NTA chromatography. Purified proteins were found to be homogeneous by using SDS-PAGE, with molecular weights estimated to be 27.9 (Glk), 50.5 (ManB), 53.0 (ManC), 42.0 (Gmd) and 36.1 (WcaG) kDa, which corresponded to theoretical values based on amino acid sequences. Protein concentrations of Glk, ManB, ManC, Gmd and WcaG were 1.32, 1.97, 2.79, 3.63 and 3.71 mg/ml (Fig. 2).

### Production of mannose-6-phosphate

**Fig. 3:**
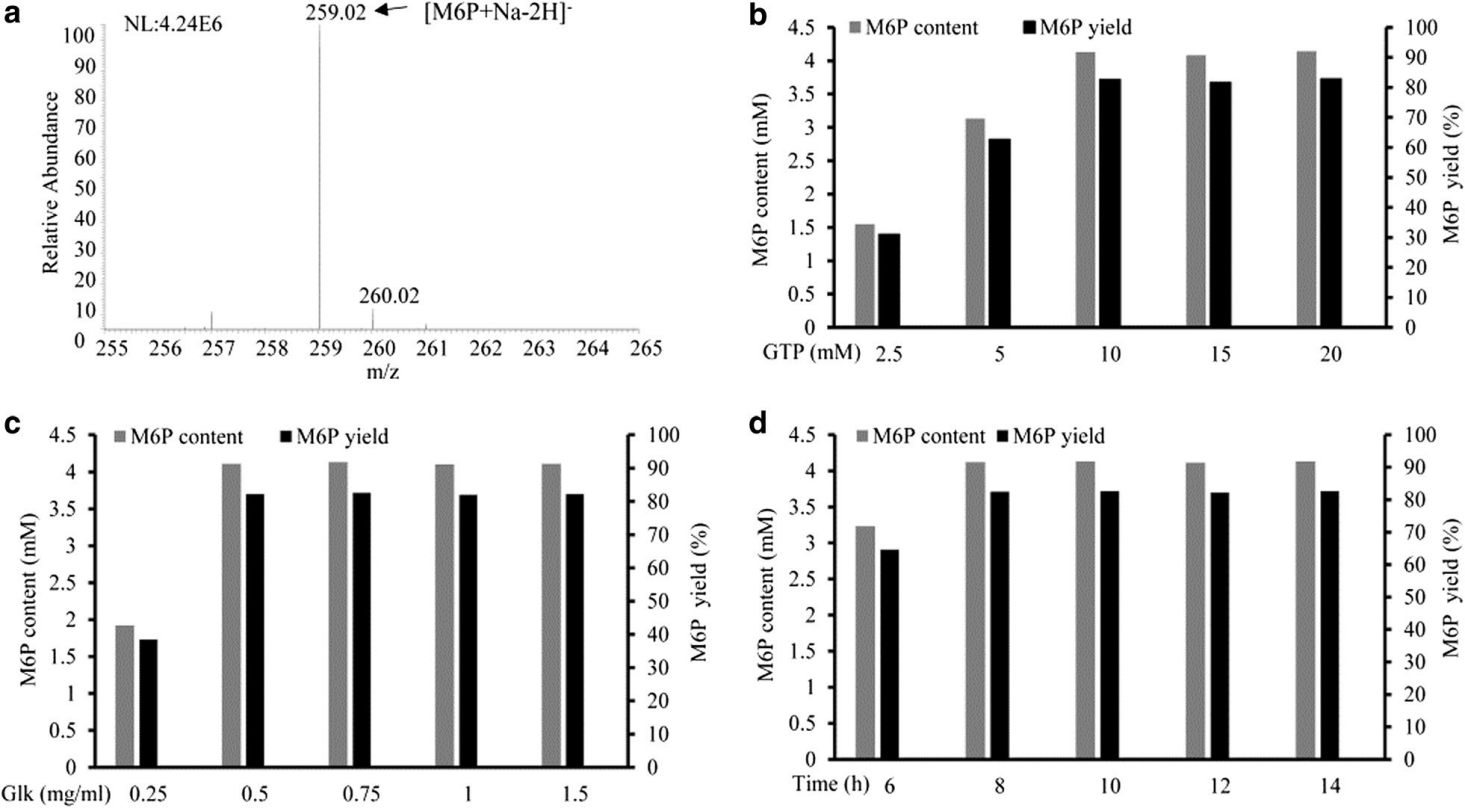
MS analysis on the production of mannose-6-phosphate (M6P) using GTP as phosphate group donor (**a**). Effects of GTP content (**b**), Glk content (**c**) and conversion time (**d**) on production of mannose-6-phosphate

According to the pathway in Fig. 1, mannose was transformed into GDP-l-fucose via mannose-6-phosphate, mannose-1-phosphate, GDP-d-mannose, and GDP-4-keto-6-deoxymannose catalyzed by glucokinase (Glk), phosphomannomutase (ManB), mannose-1-phosphate guanyltransferase (ManC), GDP-d-mannose-4,6-dehydratase (Gmd), and GDP-l-fucose synthetase (WcaG). Mannose-6-phosphate, the first intermediate in the production of GDP-l-fucose, was synthesized from mannose, catalyzed by Glk to transfer a phosphate to mannose. GTP was the phosphate donor, because it provided not only the phosphate group, but also is the cofactor for ManC production. As shown in Fig. 3a, mannose was converted into mannose-6-posphate. To improve mannose-6-phosphate production, the effect of GTP concentration, enzyme concentration, and reaction time were determined (Fig. 3b–d). Following 10 h incubation, the mannose-6-phosphate concentration was 1.2 g/l, with a conversion rate of 82.6% under optimal conditions (i.e. 10 mM GTP and 0.75 mg/ml Glk).

### Production of GDP-d-mannose

**Fig. 4:**
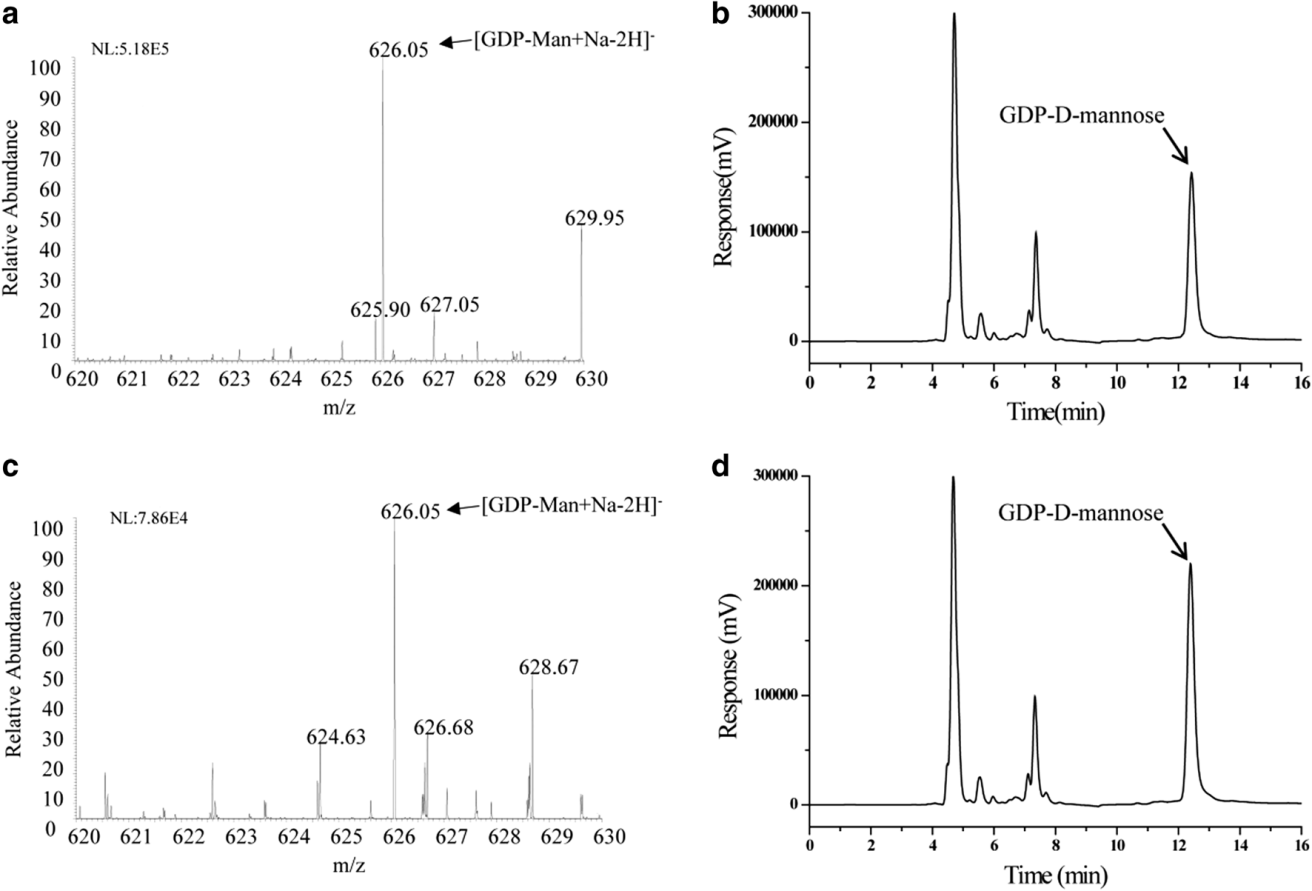
MS (**a**) and HPLC (**b**) analysis of the production of GDP-d-mannose by two-step method. Glk was firstly added into the reaction sample and incubation for 10 h, after terminating the reaction by heating, ManB and ManC were simultaneously supplied to the reaction sample for production of GDP-d-mannose. MS (**c**) and HPLC (**d**) analysis of the production of GDP-d-mannose by one-step method. Glk, ManB and ManC were simultaneously added into the reaction sample for conversion of mannose to GDP-d-mannose

GDP-d-mannose is a crucial intermediate for production of GDP-l-fucose. Here, we prepared it by conversion of mannose-6-phosphate using ManB and ManC. ManB catalyzes the reversible conversion of mannose-6-phosphate to mannose-1-phosphate. To promote the forward reaction, ManB and ManC were simultaneously added to the mannose-6-phosphate solution. Mass spectrometry (MS) analysis demonstrated a m/z of 626.05, consistent with MS data of GDP-d-mannose, illustrating that GDP-d-mannose was successfully synthesized (Fig. 4a). The yield of mannose were analyzed by HPLC, as shown in Fig. 4b, the content of GDP-d-mannose was 282.9 mg/l, with a yield of 10.4%. To optimize efficiency, Glk, ManB and ManC were simultaneously added to the mixture to initiate the reaction. Interestingly, GDP-d-mannose could still be detected by MS, and the final concentration of GDP-d-mannose reached 379.1 mg/l, with a yield of 14.6% (Fig. 4c, d). Therefore, the optimal condition for production of GDP-d-mannose was the simultaneous addition of Glk, ManB and ManC to the reaction.

### Production of GDP-l-fucose

**Fig. 5:**
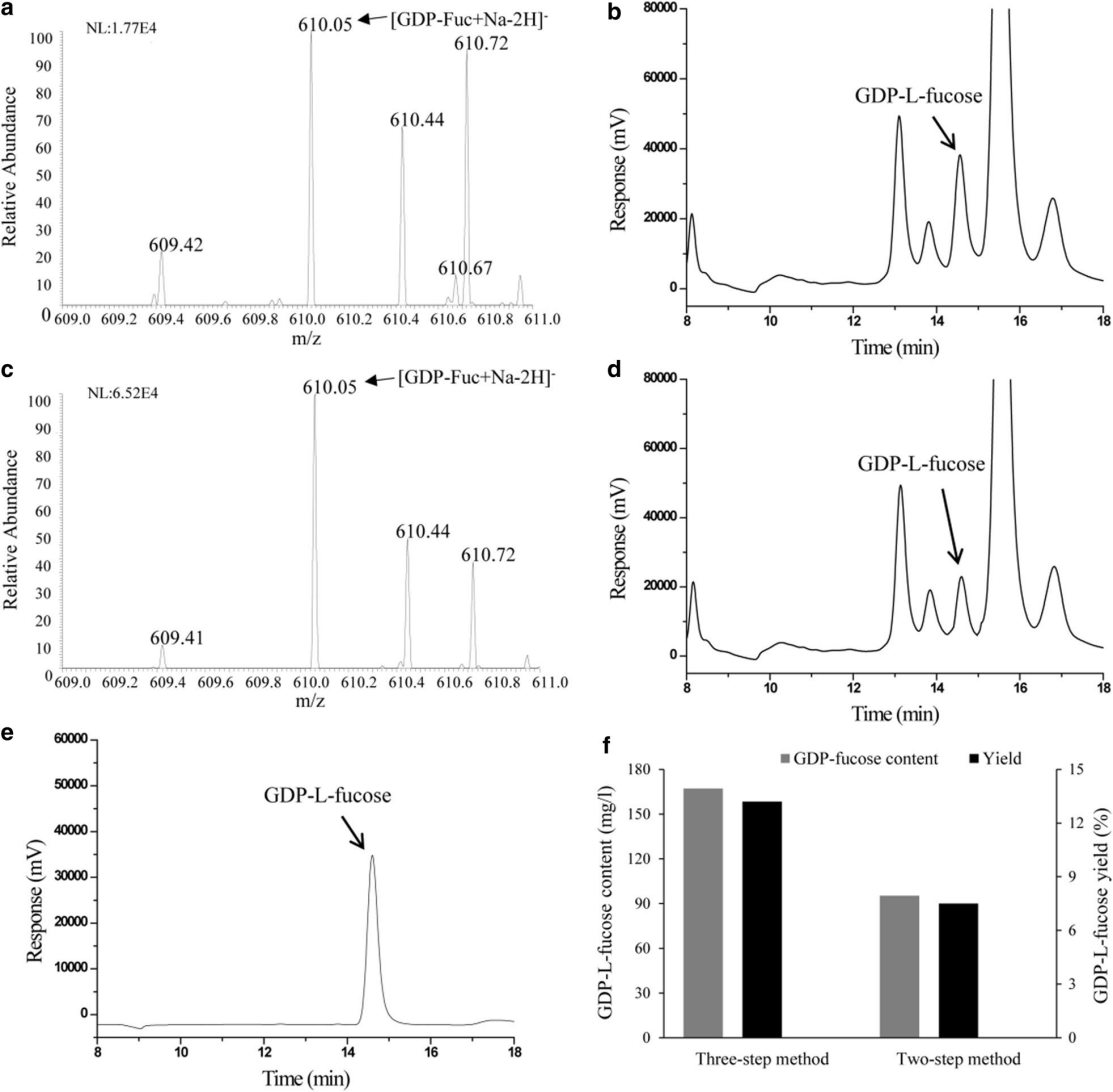
Conversion of mannose to GDP-l-fucose using three-step method and two-step method. MS [three-step method (**a**), two-step method (**c**)] and HPLC [three-step method (**b**), two-step method (**d**), standard sample (**e**)] analysis of GDP-l-fucose. The final content and yield of GDP-l-fucose (**f**)

Transformation of GDP-d-mannose to GDP-l-fucose involves GDP-d-mannose-4,6-dehydratase (Gmd) and GDP-l-fucose synthetase (WcaG). Gmd catalyzes conversion of GDP-d-Mannose to GDP-4-keto-6-deoxymannose, and WcaG converts GDP-4-keto-6-deoxymannose to the end-product GDP-l-fucose with the aid of NADPH as the reducing agent. Since Gmd is inhibited by the end-product GDP-l-fucose (Sturla et al. 1997; Bisso et al. 1999), WcaG (involved in the conversion of GDP-4-keto-6-deoxymannose to GDP-l-fucose) was added separately from Gmd to the reaction mixture to overcome feedback inhibition. Initially, Gmd was added to the reaction mixture to convert GDP-d-mannose to GDP-4-keto-6-deoxymannose. After terminating the reaction by heating, WcaG was added to produce GDP-l-fucose. The negative ion mode mass spectrum showed an intense signal at m/z 610.05, corresponding to GDP-l-fucose (calculated mass 610.05). This indicates that GDP-l-fucose can be produced by this three-step method (Fig. 5a). HPLC analysis demonstrated that the final concentration of GDP-l-fucose reached 178.6 mg/l, with a yield of 14.1% (Fig. 5b).

We next tried to simplify the conversion process by using a 2-step approach in which the reaction was composed of Glk, ManB, ManC and Gmd. Following a 12 h incubation, WcaG was added to the reaction that ran for another 2 h to produce GDP-l-fucose. Although GDP-l-fucose was produced, its yield was lower than that using the 3-step approach (Fig. 5f).

## Discussion

GDP-l-fucose is an expensive nucleotide sugar and an essential substrate for the synthesis of fucosyloligosaccharides. Chemical methods have been employed to synthesize GDP-l-fucose, but required stereospecific hydrolysis of acetobromofucose to produce the β-reducing sugar as product (Gokhale et al. 1990). Since chemical synthesis has disadvantages of being time consuming and environmentally unfriendly, it has been an impediment to industrial production of GDP-l-fucose. Microbial synthesis (preferred over chemical synthesis) can be carried out using *E. coli* or *Corynebacterium* sp. (Byun et al. 2007) have overexpressed *gmd*, *wcaG* and the gene coding for the enzyme G6PDH to produce GDP-l-fucose in *E. coli*, at a concentration of 55.2 mg/l. More recently, co-expression of *gmd*, *wcaG*, *manB* and *manC* in *C. glutamicum* has been reported, with the GDP-l-fucose content reaching 5.5 mg/g cell^−1^, about 2.4-fold higher than that of the recombinant *E. coli* overexpressing *gmd*, *wcaG*, *manB* and *manC* (Chin et al. 2013; Koizumi et al. 2000) have established large-scale production of GDP-l-fucose by using *E. coli* cells that overexpress GDP-l-fucose biosynthetic genes and *Corynebacterium ammoniagenes* that produces GTP. After 22 h starting with GMP and mannose, GDP-l-fucose was 29 mM. Consequently, engineered *Corynebacterium* sp. and *E. coli* are the primary GDP-l-fucose producers. Overexpressing GDP-l-fucose biosynthetic genes and combining engineered *E. coli* and *Corynebacterium* sp. are the two main approaches to improve the production of GDP-l-fucose. However, whole-cell fermentation increases complexity with various side reactions that hinder purification. Instead, enzymatic synthesis provides greater efficiency, faster reaction kinetics, and more facile product separation. Previously, synthesis of GDP-l-fucose was performed by using a 4-enzyme cascade in *Lactococcus lactis* using mannose-6-phosphate and GDP-mannose as substrates. However, expensive and unobtainable substrates limited its application in industrial production of GDP-l-fucose (Li et al. 2017). In our study, a 3-step process was employed to synthesize GDP-l-fucose from cheap and freely available mannose using five cell-free enzymes. Although GDP-l-fucose yield was relatively low, our method opens a new avenue to produce GDP-l-fucose. More research is required to improve the yield of GDP-l-fucose by optimizing reaction conditions and immobilizing enzymes.

In conclusion, five recombinant enzymes (Glk, ManB, ManC, Gmd and WcaG) were overexpressed in *E. coli* for the biosynthesis of GDP-l-fucose. Using these enzymes, we successfully produced GDP-l-fucose in a 3-step process. The first step involved addition of Glk, ManB and ManC to the reaction, followed by addition of Gmd and then WcaG to overcome feedback inhibition of the end-product on Gmd. GDP-L-fucose was produced at 178.6 mg/l with a conversion rate of 14.1%. This 3-step approach allows all reactions to be completed in one vessel without separation or purification of intermediates, and minimal by-products. As far as we know, this is the first report using a cell-free enzymatic synthesis of GDP-l-fucose from mannose. Further study is currently underway in our laboratory. Our research should provide the basis for industrial production of GDP-l-fucose.

## Data Availability

All data are shown in figures and tables within this article. Any material used in this study is available for research purposes upon request.

## Abbreviations

Glk: glucokinase
ManB: phosphomannomutase
ManC: mannose-1-phosphate guanyltransferase
Gmd: GDP-d-mannose-4,6-dehydratase
WcaG: GDP-l-fucose synthetase
